# Supplementary material for: Intra-cluster receptor density (IRD): A molecular switch for TNFR1 clusters’ signaling
Source: Sci Adv. 2026 Sep 2;12(36):eaed3734. doi: 10.1126/sciadv.aed3734 (PMC13537257; doi:10.1126/sciadv.aed3734)
Supplement: Supplementary file 1 [file sciadv.aed3734_sm.pdf]

Science Advances
AAAS

Supplementary Materials for

Intra-cluster receptor density (IRD): A molecular switch for TNFR1 clusters' signaling

Subhamoy Jana *et al.*

Corresponding author: Deepak Kumar Sinha, bcdks@iacs.res.in, emaildks@gmail.com

Sci. Adv. **12**, eaed3734 (2026)
DOI: 10.1126/sciadv.aed3734

The PDF file includes:

Figs. S1 to S9
Table S1
Legends for movies S1 to S3
References

Other Supplementary Material for this manuscript includes the following:

Movies S1 to S3

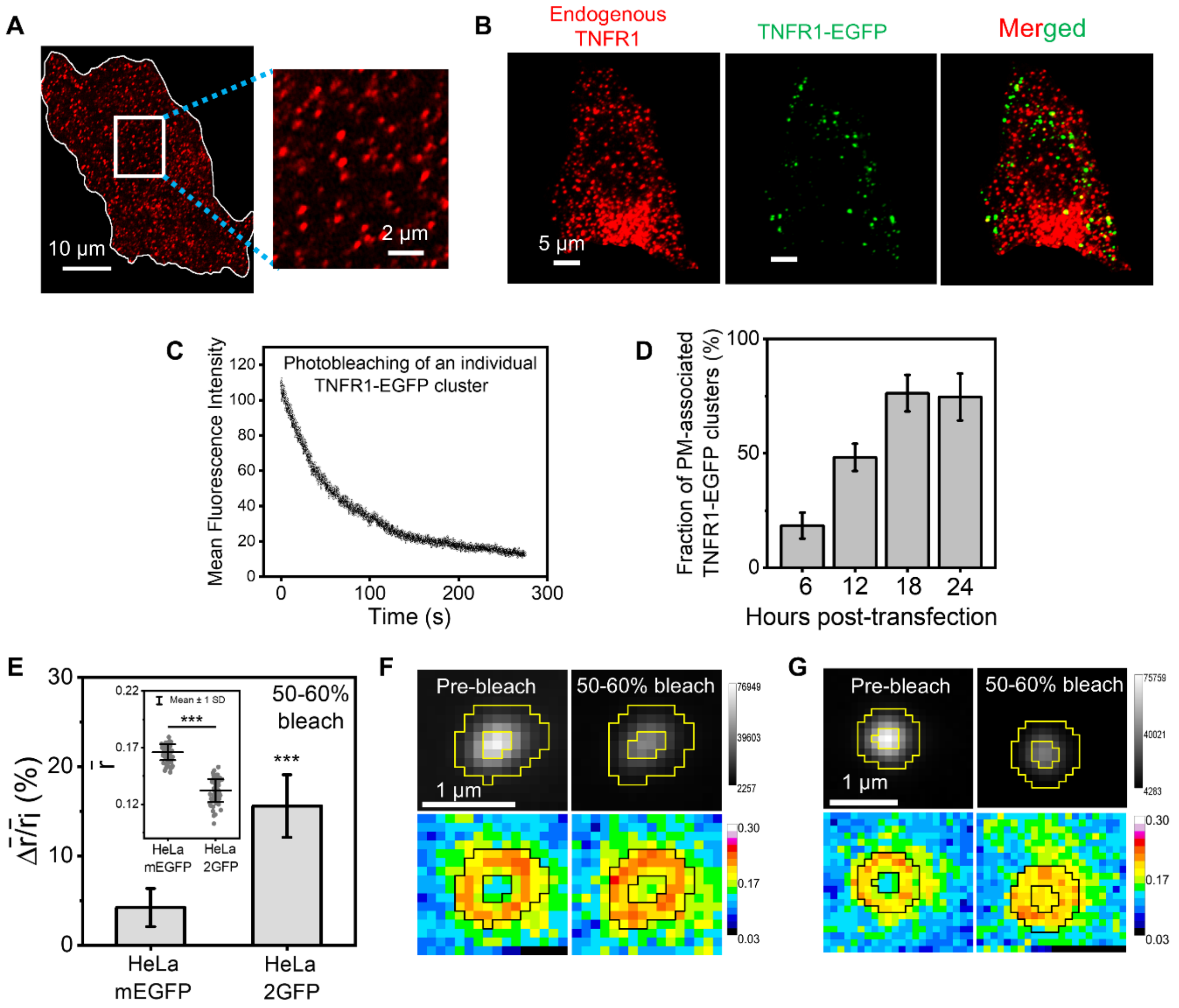

Fig. S1. Homo-FRET analysis of TNFR1-EGFP clusters reveals spatial organization and dynamic remodeling of TNFR1 in HeLa cells.

(**A**) Representative Airyscan image of a HeLa cell immunostained for TNFR1 (red, left), with a magnified view highlighting endogenous TNFR1 clusters (right). (**B**) Representative confocal images of a HeLa cell expressing TNFR1-EGFP (green, middle) and immunostained with anti-TNFR1 antibody (red, left); merged image (right) shows their colocalization. *This figure is reused from Fig. 1B to show different channels separately*. (**C**) Mean fluorescence intensity of a single TNFR1-EGFP cluster plotted over time during photobleaching. (**D**) Quantification of the fraction of plasma membrane (PM)–associated TNFR1-EGFP clusters over time post-transfection (means ± SD; 6 cells, 2 experiments). (**E**) Percentage change in relative mean fluorescence anisotropy ($\Delta\bar{r}/\bar{r}_i$) of mEGFP and 2EGFP overexpressed HeLa cells upon photobleaching (40 cells, 2 experiments). Here, $\Delta\bar{r} = \bar{r}_f - \bar{r}_i$ where $\bar{r}_f$ and $\bar{r}_i$ represent post- and pre-bleach mean anisotropy, respectively. The inset shows the initial mean

fluorescence anisotropy ($\bar{r}$) of mEGFP- and 2EGFP-overexpressed HeLa cells (70 cells, 3 experiments). (**F**) Representative fluorescence intensity (top) and corresponding fluorescence anisotropy (bottom) maps of a TNFR1-EGFP cluster before and after photobleaching. (**G**) Representative TNFR1-EGFP cluster showing fluorescence intensity (top) and corresponding fluorescence anisotropy (bottom) maps before and after photobleaching, illustrating uniform fluorescence anisotropy across both the core and the rim of the cluster upon photobleaching. Statistical significance was determined by an unpaired two-tailed Student's *t*-test (E) and is indicated in the figures as follows: $*P < 0.05$; $**P < 0.01$; $***P < 0.001$; ns, not significant. Scale bars as indicated.

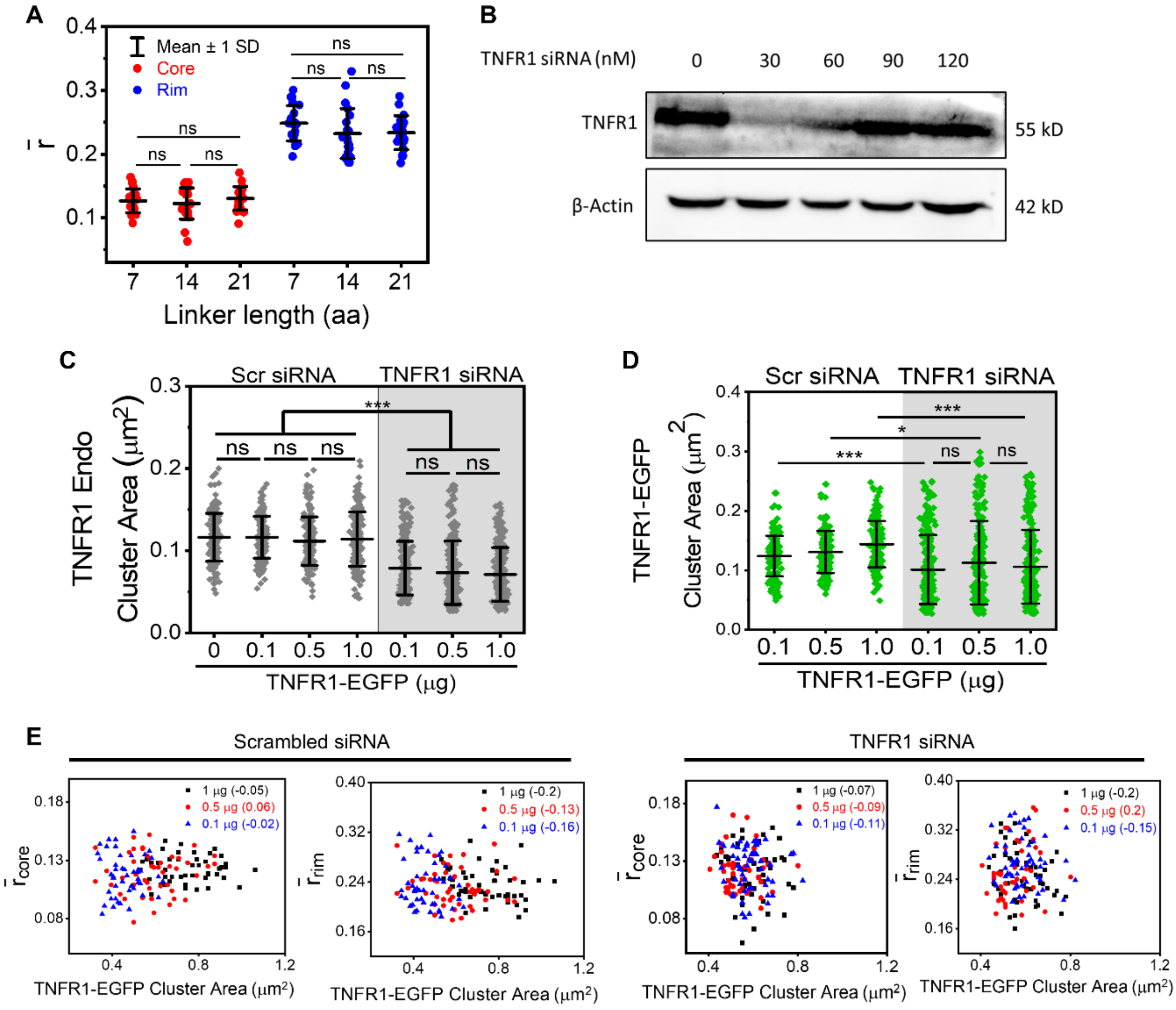

Fig. S2. TNFR1 expression level and IRD influence cluster area and organization.

(A) Mean fluorescence anisotropy ($\bar{r}$) of the core and rim regions of TNFR1-EGFP clusters in HeLa cells expressing TNFR1-EGFP constructs with different linker lengths (21 clusters, 3 cells, 2 experiments per condition). **(B)** Immunoblot showing TNFR1 expression in HeLa cells treated with increasing doses of TNFR1 siRNA. β-Actin was used as a loading control. **(C)** Quantification of the area of endogenous TNFR1 clusters in TNFR1-EGFP–transfected HeLa cells pre-treated with scrambled-siRNA or TNFR1-siRNA, as acquired by Airyscan imaging (≥170 clusters, 8 cells, 3 experiments per condition). **(D)** Quantification of the area of TNFR1-EGFP clusters in TNFR1-EGFP–transfected HeLa cells pre-treated with scrambled-siRNA or TNFR1-siRNA, as acquired by Airyscan imaging (≥170 clusters, 8 cells, 3 experiments per condition). **(E)** Scatter plots showing the relationship between TNFR1-EGFP cluster area and mean fluorescence anisotropy of the core ($\bar{r}_{core}$) and rim ($\bar{r}_{rim}$) of TNFR1-EGFP clusters in HeLa cells transfected with scrambled-siRNA or TNFR1-siRNA, and co-transfected with

increasing doses of TNFR1-EGFP plasmid (45 clusters, 6 cells, 3 experiments per condition). Pearson's correlation coefficients are indicated in the figure for the corresponding TNFR1-EGFP doses. Statistical significance was determined by one-way ANOVA (A, C, D) and is indicated in the figures as follows: $*P < 0.05$; $**P < 0.01$; $***P < 0.001$; ns, not significant.

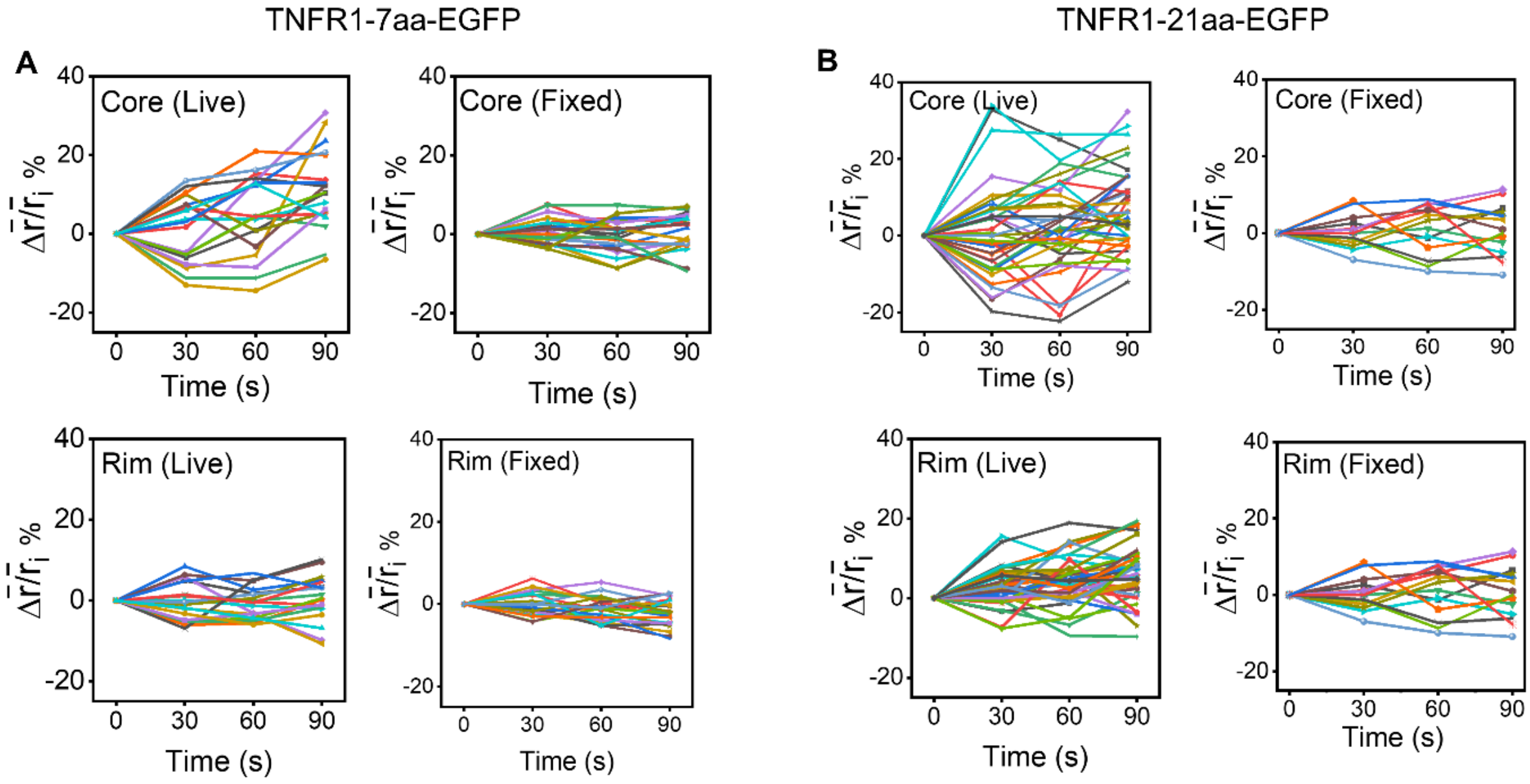

Fig. S3. Temporal dynamics of fluorescence anisotropy in live and fixed TNFR1-EGFP clusters with variable linker lengths.

(A) Percentage change in relative mean fluorescence anisotropy ($\Delta \bar{r}/\bar{r}_i$), where $\Delta\bar{r} = \bar{r}(t) - \bar{r}_i$, for the core (top) and rim (bottom) plotted as a function of time (t) in live (19 clusters) and fixed (21 clusters) HeLa cells expressing TNFR1-7aa-EGFP. Here, $\bar{r}_i$ represents the initial mean anisotropy (t = 0), and $\bar{r}(t)$ denotes the mean anisotropy at time t (4 cells, 2 experiments). (B) Percentage change in relative mean fluorescence anisotropy ($\Delta \bar{r}/\bar{r}_i$) for the core (top) and rim (bottom) regions plotted as a function of time (t) in live (35 clusters) and fixed (15 clusters) HeLa cells expressing TNFR1-21aa-EGFP (4 cells, 2 experiments).

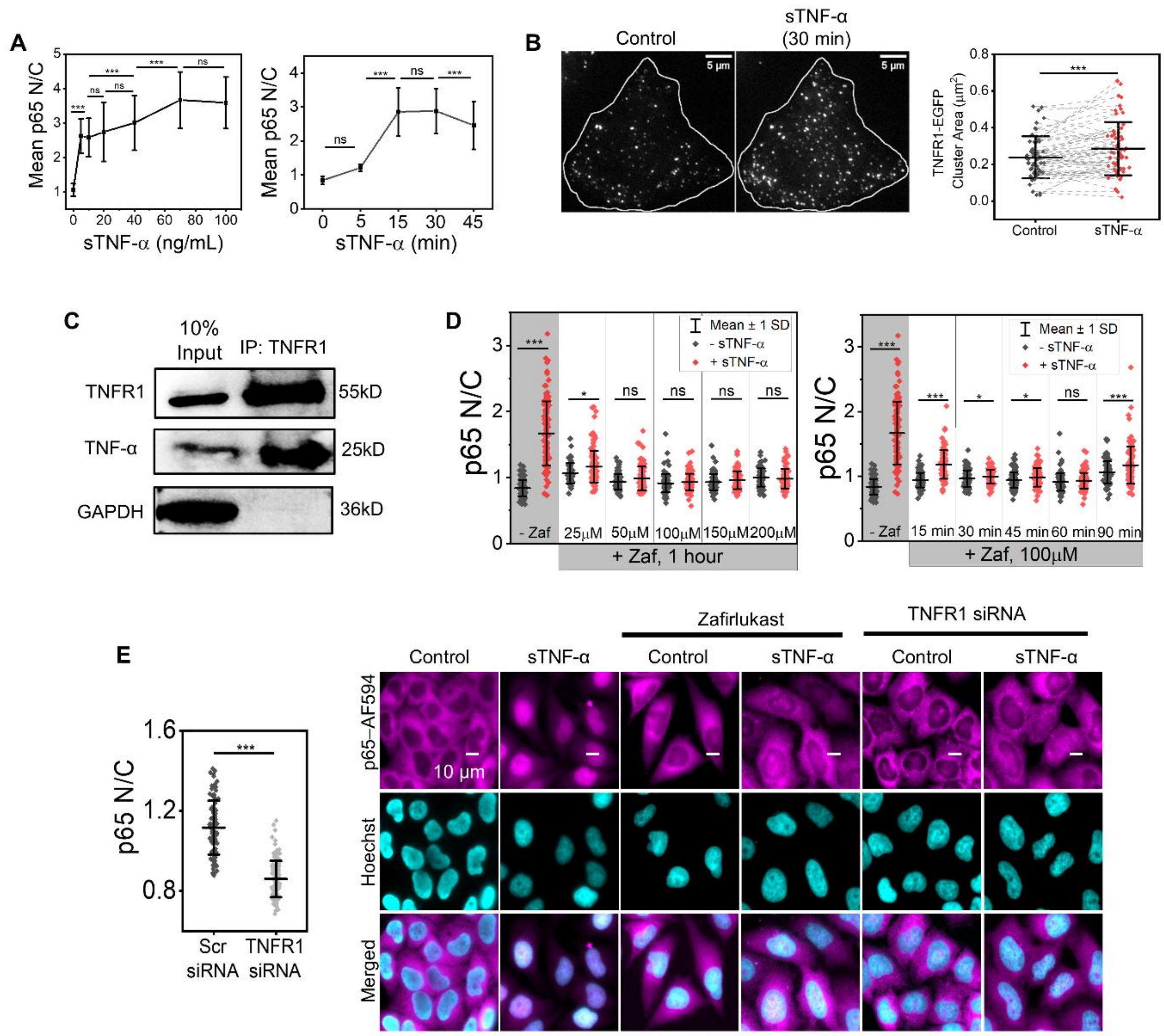

Fig. S4. Differential modulation of TNFR1-mediated NF-κB activation by sTNF-α, zafirlukast, and receptor expression levels.

(**A**) Quantification of the p65 N/C mean fluorescence intensity ratio in HeLa cells treated with increasing concentrations of sTNF-α for 30 min (left), and with 20 ng/mL sTNF-α for varying durations (right) (90 cells, 3 experiments; mean ± SD). (**B**) Representative TIRF images of TNFR1-EGFP-expressing HeLa cells before (Control) and after stimulation with sTNF-α. Quantification of the area of similar TNFR1-EGFP clusters before (Control) and after sTNF-α stimulation is shown (60 clusters, 6 cells, 3 experiments). (**C**) Immunoblot analysis of TNFR1 and TNF-α in HeLa cell lysates subjected to immunoprecipitation (IP) using an anti-TNFR1 antibody. Both input and IP: TNFR1 samples show positive bands for TNFR1 and TNF-α, while GAPDH served as a negative control. Representative of two independent experiments. (**D**) Quantification of p65 N/C ratio in HeLa cells treated with increasing concentrations of

zafirlukast in the presence or absence of sTNF-α (left), and in cells treated with 100 μM Zaf for varying durations with or without sTNF-α stimulation (right) (≥63 cells, 3 experiments per condition). (**E**) Quantification of p65 N/C mean fluorescence intensity ratio in HeLa cells transfected with scrambled or TNFR1-siRNA (≥109 cells, 3 experiments per condition; left). Representative immunofluorescence images (right) show p65 (magenta, top) in HeLa cells treated with sTNF-α under control, zafirlukast-treated, and TNFR1-siRNA conditions. DNA was counterstained with Hoechst 33342 (cyan, middle); merged images are shown in the lower panel. Statistical significance was determined using one-way ANOVA (A), paired two-tailed Student's *t*-test (B), or unpaired two-tailed Student's *t*-test (C, D), and is indicated in the figures as follows: $*P < 0.05$; $**P < 0.01$; $***P < 0.001$; ns, not significant. Scale bars as indicated.

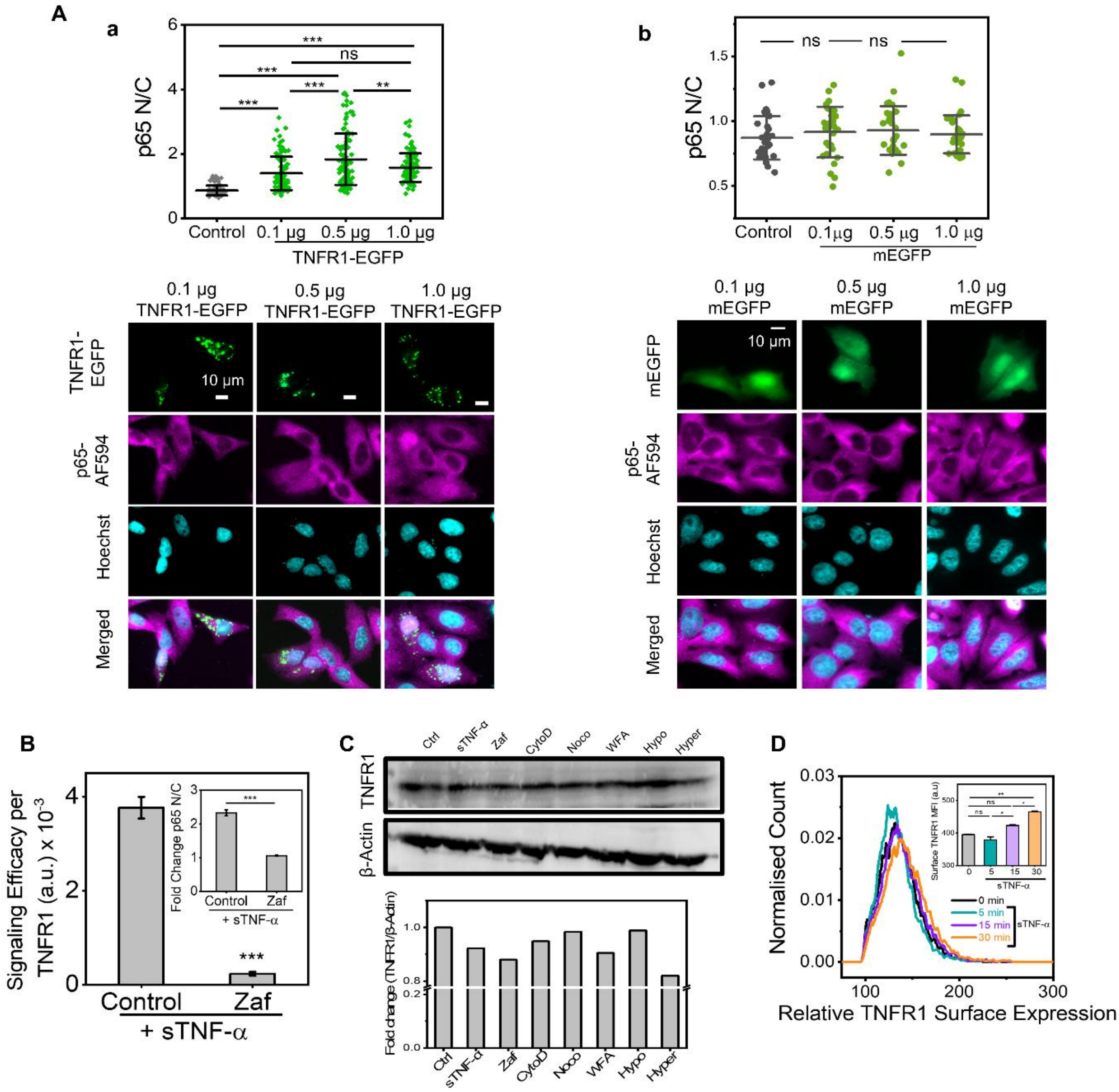

Fig. S5. Innate levels of TNFR1 expression influence the basal activation of NF-κB.

(**A**) Quantification of endogenous p65 N/C mean fluorescence intensity ratio in HeLa cells transfected with increasing doses of TNFR1-EGFP (Aa, top), and mEGFP (Ab, top) (TNFR1-EGFP: ≥104 cells, 3 experiments per condition; EGFP: 30 cells, 2 experiments per condition). Representative immunofluorescence images of TNFR1-EGFP (Aa, bottom: green) and mEGFP (Ab, bottom: green) HeLa cells for p65 (magenta), nuclei stained with Hoechst 33342 (cyan), and merged channels. (**B**) Signaling efficacy of TNFR1 in control and zafirlukast-treated HeLa cells in response to sTNF-α. The inset shows fold changes in p65 N/C ratio normalized to their respective basal (unstimulated) levels (mean ± SD; 90 cells, 3 experiments per condition). (**C**) Immunoblot analysis of total TNFR1 protein levels in HeLa cells under various treatments —

control (Ctrl), sTNF-α (20 ng/mL, 5 min), zafirlukast (Zaf, 100 μM, 1 h), cytochalasin D (CytoD, 2 μM, 1 h), nocodazole (Noco, 18 μM, 2 h), withaferin A (WFA, 2 μM, 4 h), hypotonic (Hypo, 5 min), and hypertonic (Hyper, 10 min) conditions. β-Actin served as a loading control. Densitometric quantification of fold change in TNFR1 expression normalized to β-Actin is shown below. (**D**) Flow cytometric analysis of surface TNFR1 expression in control (0 min) and sTNF-α-treated (5–30 min) HeLa cells. Quantification of median fluorescence intensity (MFI) for each condition is shown in the inset (2 experiments). Statistical significance was determined using one-way ANOVA (A, D) or unpaired two-tailed Student's *t*-test (B) and is indicated in the figures as follows: ∗P < 0.05; ∗∗P < 0.01; ∗∗∗P < 0.001; ns, not significant. Scale bars as indicated.

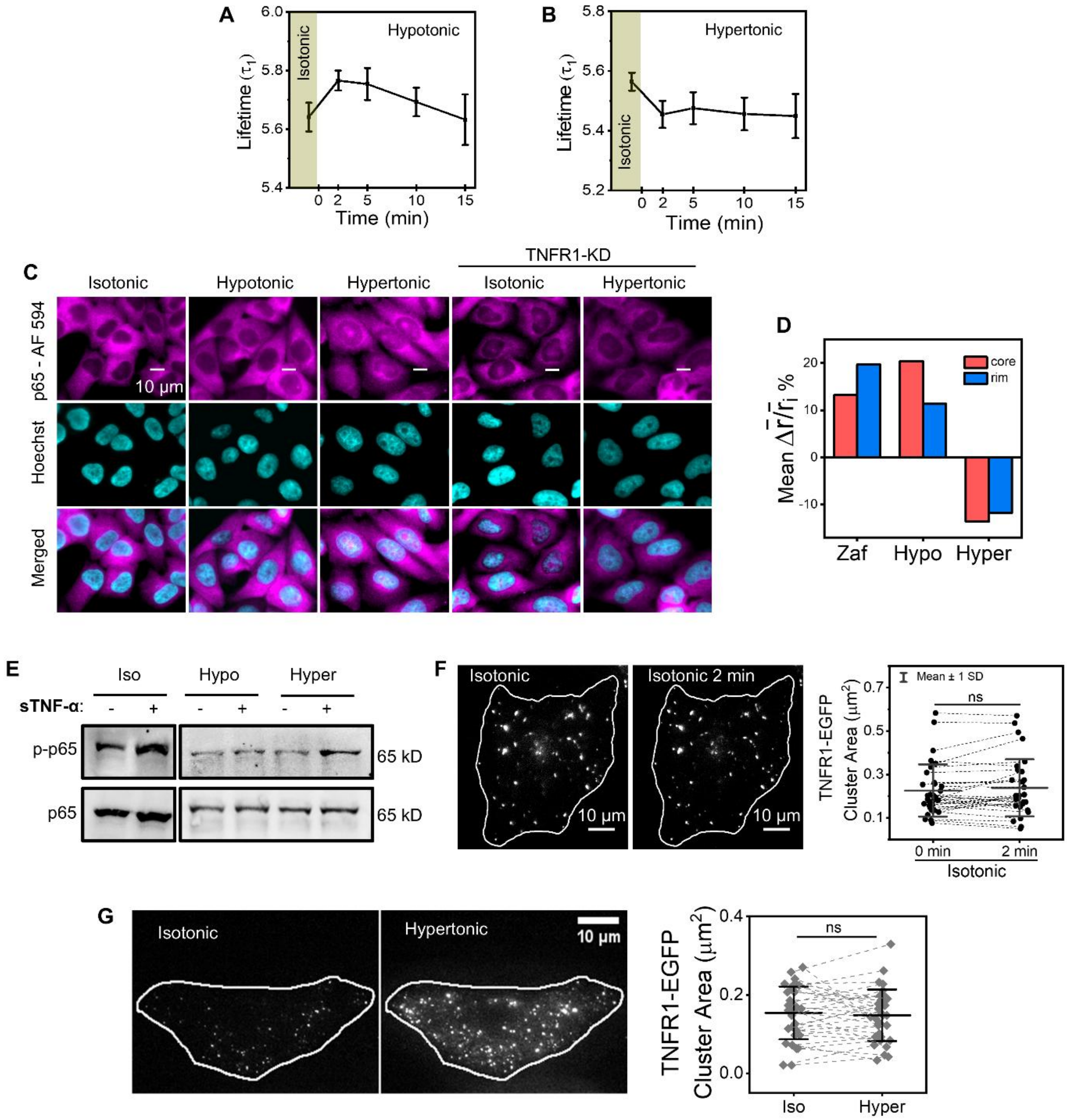

Fig. S6. Membrane tension regulates TNFR1 IRD and downstream NF-κB signaling in HeLa cells.

(**A** and **B**) Fluorescence lifetime (τ_1) measurements of Flipper-TR in HeLa cells under hypotonic (A) and hypertonic (B) conditions over 15 minutes. (mean ± SD; 15 cells, 2 experiments). (**C**) Representative immunofluorescence images of HeLa cells showing p65 localization (magenta, top) under isotonic, hypotonic, and hypertonic conditions in control and TNFR1-knockdown cells. Nuclei were counterstained with Hoechst 33342 (cyan, middle), and merged images are shown in the bottom panel. (**D**) Percentage change in relative mean

fluorescence anisotropy ($\Delta\,\bar{r}/\bar{r}_i$) of the core and rim regions of TNFR1-EGFP clusters in HeLa cells treated with zafirlukast, hypotonic, or hypertonic conditions, normalized to respective controls. Here, $\Delta\bar{r} = \bar{r}_f - \bar{r}_i$ where $\bar{r}_f$ and $\bar{r}_i$ represent the final and initial mean anisotropy for each treatment, respectively (60 cells, 3 experiments per condition). (**E**) Immunoblot analysis of Ser536 phosphorylated p65 (p-p65) levels in HeLa cells subjected to hypotonic or hypertonic conditions, followed by sTNF-α stimulation, compared to isotonic controls. Total p65 served as a loading control. Representative of two independent experiments. (**F**) Representative TIRF images of TNFR1-EGFP–expressing HeLa cells under isotonic conditions (left). Quantification of the area of the same TNFR1-EGFP clusters before and after 2 minutes is shown (right) (32 clusters, 4 cells, 2 experiments). (**G**) Representative TIRF images of TNFR1-EGFP–transfected HeLa cells before and after 10 minutes of hypertonic condition (left). Quantification of the area of the same TNFR1-EGFP clusters before (Iso) and after hypertonic (Hyper) condition is shown (right) (34 clusters, 4 cells, 2 experiments). Statistical significance was determined by paired two-tailed Student's *t*-test (F, G) and is indicated in the figures as follows: ∗P < 0.05; ∗∗P < 0.01; ∗∗∗P < 0.001; ns, not significant. Scale bars as indicated.

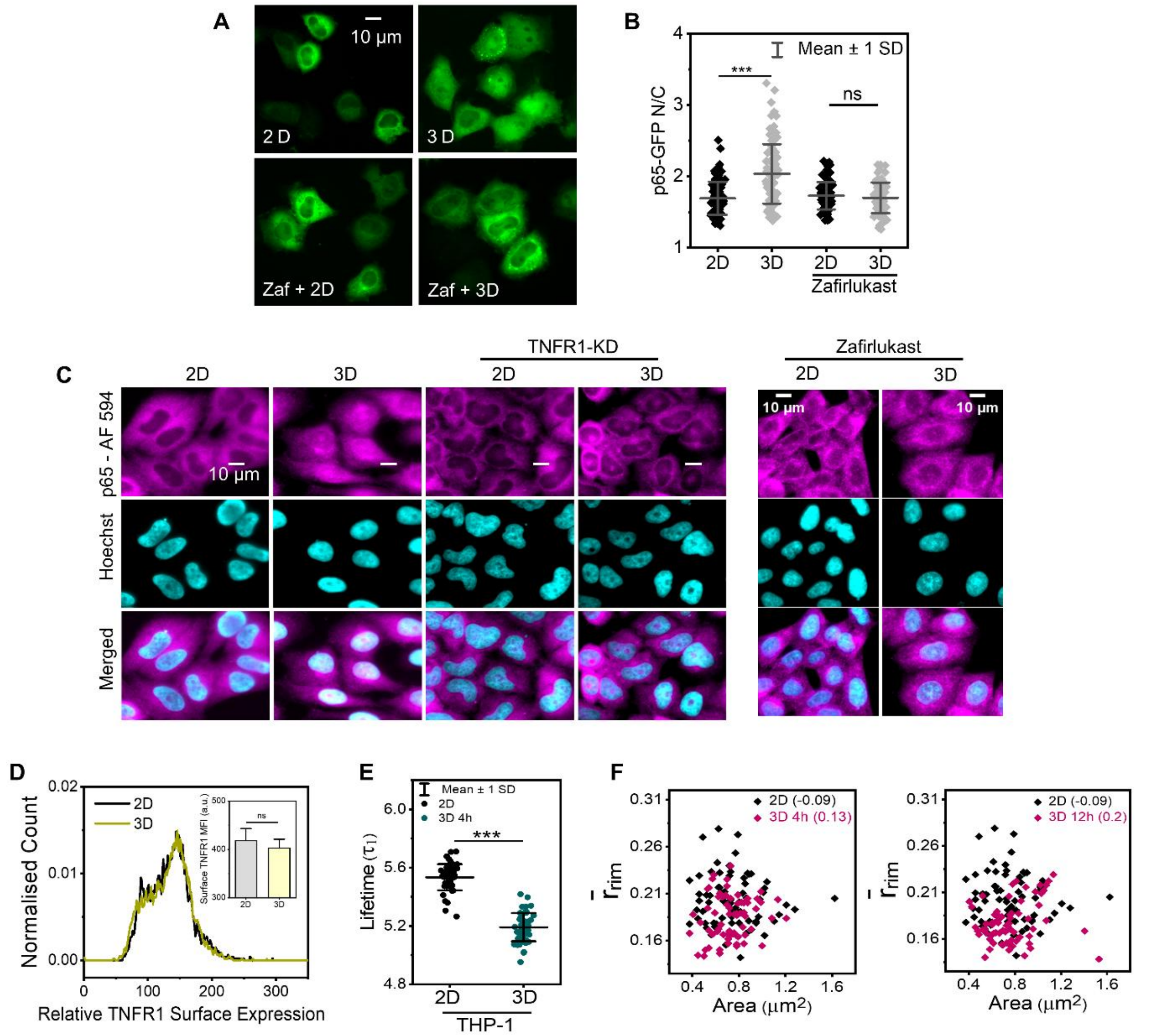

Fig. S7. TNFR1 signaling and membrane tension are modulated by a 3D gel-like microenvironment.

(A) Live-cell imaging of representative HeLa cells expressing p65-GFP cultured in 2D and 3D under untreated and zafirlukast-treated conditions. (B) p65-EGFP N/C in live HeLa cells under the indicated conditions (≥96 cells, 3 experiments per condition). (C) Representative immunofluorescence images of p65 (magenta, top) in HeLa cells cultured in 2D or 3D under control, TNFR1-knockdown, and zafirlukast-treated conditions. Nuclei were counterstained with Hoechst 33342 (cyan, middle), and merged images are shown in the bottom panel. (D) Surface expression of endogenous TNFR1 in HeLa cells cultured in 2D and 3D conditions, analyzed by flow cytometry. The inset shows the median fluorescence intensity (MFI) for the 2D and 3D conditions. (E) Fluorescence lifetime (τ_1) of the membrane tension probe Flipper-TR in THP-1 cells cultured in 2D and 3D conditions (50 cells, 3 experiments per condition).

(F) Scatter plots showing TNFR1-EGFP cluster area versus mean fluorescence anisotropy of the rim ($\bar{r}_{rim}$) of TNFR1-EGFP clusters in HeLa cells cultured in 2D and 3D conditions. Pearson's correlation coefficients are indicated for each condition (70 clusters, 9 cells, 3 experiments per condition). Statistical significance was determined by one-way ANOVA (B) or unpaired two-tailed Student's *t*-test (D, E), as indicated in the figures as follows: $*P < 0.05$; $**P < 0.01$; $***P < 0.001$; ns, not significant. Scale bars as indicated.

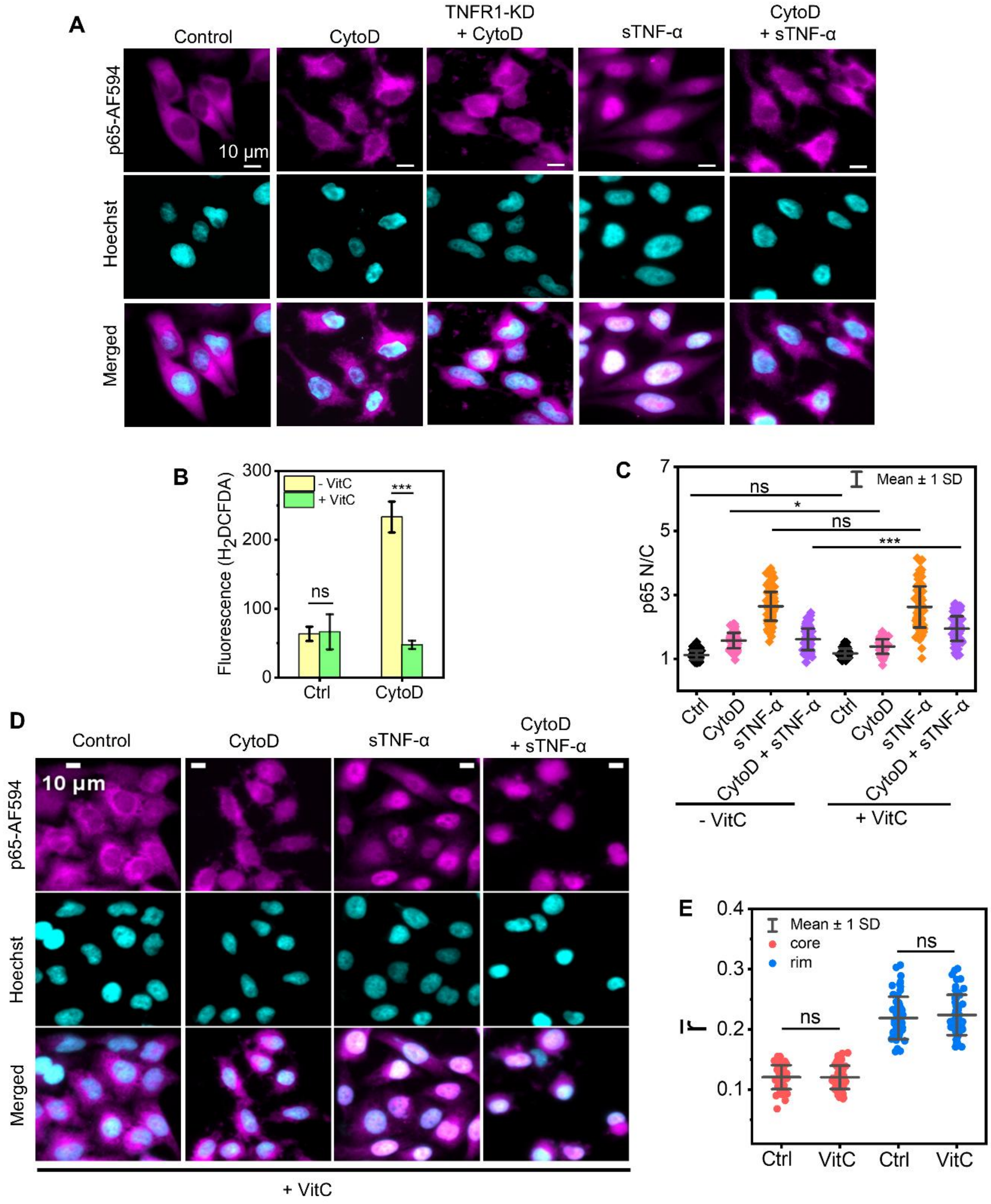

Fig. S8. Impact of actin disruption on TNFR1 signaling and cluster organization.

(**A**) Representative immunofluorescence images of HeLa cells stained for p65 (magenta, top) under cytochalasin D treatment in control and TNFR1-knockdown cells, and following sTNF-α stimulation in the presence or absence of Cytochalasin D. Nuclei were counterstained with Hoechst 33342 (cyan, middle), and merged images are shown in the bottom panel. (**B**) Quantification of mean H_2DCFDA fluorescence intensity (a.u.) in control and CytoD-treated

HeLa cells, with or without Vitamin C (VitC) pre-treatment. (**C**) p65 N/C mean fluorescence intensity ratio in HeLa cells under control, CytoD-treated, sTNF-α-treated, and combined CytoD + sTNF-α-treated conditions, with or without VitC pre-treatment (mean ± SD; ≥85 cells, 3 experiments per condition). (**D**) Representative immunofluorescence images of p65 (magenta, top) in HeLa cells pre-treated with VitC under control, CytoD-treated, sTNF-α-treated, and combined CytoD + sTNF-α-treated conditions. Nuclei were counterstained with Hoechst 33342 (cyan, middle), and merged images are shown in the bottom panel. (**E**) Mean fluorescence anisotropy ($\bar{r}$) of the core and rim of TNFR1-EGFP clusters in control and VitC-treated HeLa cells (53 clusters, 6 cells, 2 experiments per condition). Statistical significance between datasets was determined by unpaired, two-tailed Student's *t*-test (B, E) or one-way ANOVA (C) and is indicated in the figures as follows: ∗P < 0.05; ∗∗P < 0.01; ∗∗∗P < 0.001; ns, not significant. Scale bars as indicated.

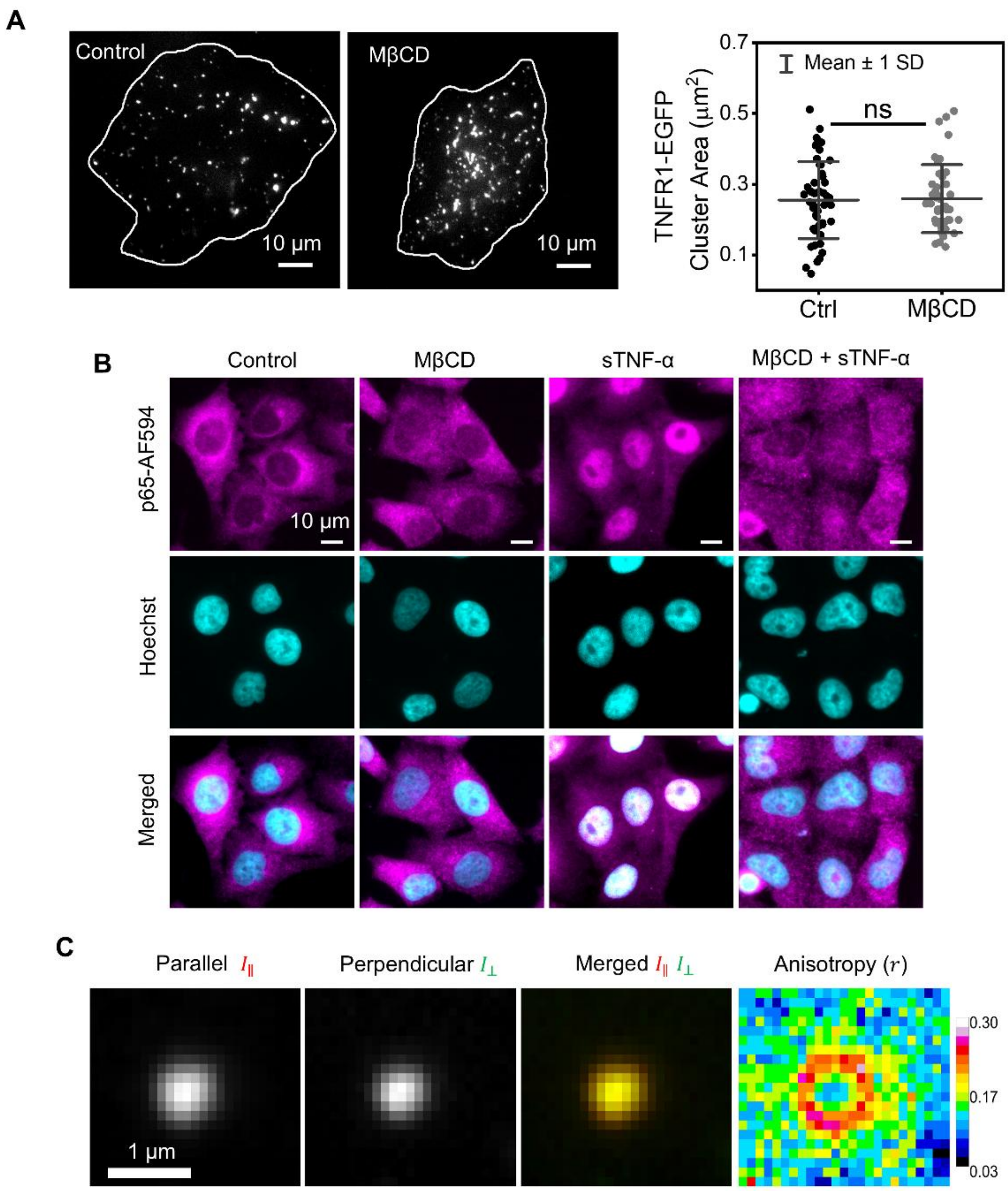

Fig. S9. Effects of membrane cholesterol depletion on TNFR1 clustering and signaling.

(**A**) Representative TIRF images (left) of a TNFR1–EGFP–expressing HeLa cell before and after treatment with methyl-β-cyclodextrin. Quantification of immobile TNFR1–EGFP cluster area before and after MβCD treatment is shown (right) (≥43 clusters, 6 cells, 2 experiments per condition). (**B**) Representative immunofluorescence images of p65 (magenta, top) in HeLa cells under control, MβCD-treated, sTNF-α-treated, and combined MβCD + sTNF-α-treated conditions. Nuclei were counterstained with Hoechst 33342 (cyan, middle), and merged images are shown in the bottom panel. (**C**) Representative epifluorescence images of a TNFR1–EGFP cluster in parallel (left) and perpendicular (second left) polarization channels, with the merged image (second right) and the corresponding anisotropy map (right). Statistical significance was

determined by unpaired two-tailed Student's *t*-test (A) and is indicated in the figures as follows: $*P < 0.05$; $**P < 0.01$; $***P < 0.001$; ns, not significant. Scale bars as indicated.

Table S1. Summary of inhibitors that modulate TNFR1 clustering or signaling, along with their mechanisms of action and corresponding references.

Inhibitor	Target / Mode of Action	Reference
**SPD304**	Binds to TNF-α and dissociates its trimeric structure, thus preventing TNFR1 activation.	(74, 75)
**IW927**	Directly binds TNFR1 and blocks TNF-α binding, thereby disrupting receptor activation.	(76)
**Zafirlukast**	Inhibits PLAD-mediated dimerization of TNFR1, thus impairing receptor clustering and signaling.	(33, 34)
**UCB-9260**	Stabilizes an asymmetric TNF-α trimer, preventing effective TNFR1 engagement for downstream signal activation.	(77)
**F002**	Cavity-induced allosteric modifier (CIAM) that prevents TNF-α binding to TNFR1 and thereby blocks subsequent pathway activation.	(*78*)
**SGT11, C7**	Bind to TNFR1 allosteric sites; inhibit TNF-α–induced NF-κB signaling.	(79)
**Suramin, Evans Blue, Trypan Blue**	Interfere with TNF-α trimerization, indirectly preventing TNFR1 activation.	(80, 81)
**Cyclic peptides**	Selectively bind TNFR1 and inhibit receptor-ligand interaction.	(82)

Movie S1.

3D-rendered images of a HeLa cell viewed from different orientations, showing the distribution of TNFR1-EGFP clusters (green). The plasma membrane is stained with CellMask Orange (orange).

Movie S2.

Time-lapse imaging of TNFR1-EGFP clusters in a HeLa cell, comprising 20 frames captured at 2-second intervals. The movie shows intracellular mobile clusters (yellow circle) and plasma membrane–associated clusters that remain relatively immobile (red circle).

Movie S3.

Time-lapse imaging of TNFR1-EGFP clusters in untreated control (left) and dynasore-treated (80 μM, 50 min, right) HeLa cells. Imaging was performed continuously for 600 frames with an acquisition rate of 200 ms per frame.

REFERENCES

1. H. T. Idriss, J. H. Naismith, TNFα and the TNF receptor superfamily: Structure-function relationship(s). *Microsc. Res. Tech.* **50**, 184–195 (2000).

2. É. S. Vanamee, D. L. Faustman, Structural principles of tumor necrosis factor superfamily signaling. *Sci. Signal.* **11**, eaao4910 (2018).

3. M. Croft, W. Duan, H. Choi, S.-Y. Eun, S. Madireddi, A. Mehta, TNF superfamily in inflammatory disease: Translating basic insights. *Trends Immunol.* **33**, 144–152 (2012).

4. G. van Loo, M. J. M. Bertrand, Death by TNF: A road to inflammation. *Nat. Rev. Immunol.* **23**, 289–303 (2023).

5. Y. Takei, R. Laskey, Tumor Necrosis factor α Regulates responses to nerve growth factor, promoting neural cell survival but suppressing differentiation of neuroblastoma cells. *Mol. Biol. Cell* **19**, 855–864 (2008).

6. A. Bhardwaj, B. B. Aggarwal, Receptor-mediated choreography of life and death. *J. Clin. Immunol.* **23**, 317–332 (2003).

7. O. Micheau, J. Tschopp, Induction of TNF receptor I-mediated apoptosis via two sequential signaling complexes. *Cell* **114**, 181–190 (2003).

8. S. Yang, C. Xie, Y. Chen, J. Wang, X. Chen, Z. Lu, R. R. June, S. G. Zheng, Differential roles of TNFα-TNFR1 and TNFα-TNFR2 in the differentiation and function of CD4+Foxp3+ induced Treg cells in vitro and in vivo periphery in autoimmune diseases. *Cell Death Dis.* **10**, 27 (2019).

9. J. D. Webster, D. Vucic, The balance of TNF Mediated pathways regulates inflammatory cell death signaling in healthy and diseased tissues. *Front. Cell Dev. Biol.* **8**, 365 (2020).

10. G. Chen, D. V. Goeddel, TNF-R1 signaling: A beautiful pathway. *Science* **296**, 1634–1635 (2002).

11. R. A. Smith, C. Baglioni, The active form of tumor necrosis factor is a trimer. *J. Biol. Chem.* **262**, 6951–6954 (1987).

12. J. H. Naismith, B. J. Brandhuber, T. Q. Devine, S. R. Sprang, Seeing double: Crystal structures of the type I TNF receptor. *J. Mol. Recognit.* **9**, 113–117 (1996).

13. D. W. Banner, A. D'Arcy, W. Janes, R. Gentz, H.-J. Schoenfeld, C. Broger, H. Loetscher, W. Lesslauer, Crystal structure of the soluble human 55 kd TNF receptor-human TNFβ complex: Implications for TNF receptor activation. *Cell* **73**, 431–445 (1993).

14. C. Karathanasis, J. Medler, F. Fricke, S. Smith, S. Malkusch, D. Widera, S. Fulda, H. Wajant, S. J. L. van Wijk, I. Dikic, M. Heilemann, Single-molecule imaging reveals the oligomeric state of functional TNFα-induced plasma membrane TNFR1 clusters in cells. *Sci. Signal.* **13**, 5647 (2020).

15. K. Kucka, H. Wajant, Receptor oligomerization and its relevance for signaling by receptors of the tumor necrosis factor receptor superfamily. *Front. Cell Dev. Biol.* **8**, 615141 (2021).

16. F. Fricke, S. Malkusch, G. Wangorsch, J. F. Greiner, B. Kaltschmidt, C. Kaltschmidt, D. Widera, T. Dandekar, M. Heilemann, Quantitative single-molecule localization microscopy combined with rule-based modeling reveals ligand-induced TNF-R1 reorganization toward higher-order oligomers. *Histochem. Cell Biol.* **142**, 91–101 (2014).

17. F. K.-M. Chan, H. J. Chun, L. Zheng, R. M. Siegel, K. L. Bui, M. J. Lenardo, A domain in TNF receptors that mediates ligand-independent receptor assembly and signaling. *Science* **288**, 2351–2354 (2000).

18. É. S. Vanamee, D. L. Faustman, The benefits of clustering in TNF receptor superfamily signaling. *Front. Immunol.* **14**, 1225704 (2023).

19. C. S. Lim, J. Lee, J. W. Kim, J.-O. Lee, Highly ordered clustering of TNFα and BAFF ligand-receptor-intracellular adaptor complexes on a lipid membrane. *Nat. Commun.* **16**, 5551 (2025).

20. M. Branschädel, A. Aird, A. Zappe, C. Tietz, A. Krippner-Heidenreich, P. Scheurich, Dual function of cysteine rich domain (CRD) 1 of TNF receptor type 1: Conformational stabilization of CRD2 and control of receptor responsiveness. *Cell. Signal.* **22**, 404–414 (2010).

21. L. Zhao, Q. Fu, L. Pan, A. Piai, J. J. Chou, The diversity and similarity of transmembrane trimerization of TNF receptors. *Front. Cell Dev. Biol.* **8**, 569684 (2020).

22. P. E. Morton, C. Perrin, J. Levitt, D. R. Matthews, R. J. Marsh, R. Pike, D. Mcmillan, A. Maloney, S. Poland, S. Ameer-Beg, M. Parsons, TNFR1 membrane reorganization promotes distinct modes of TNFα signaling. *Sci. Signal.* **12**, eaaw2418 (2019).

23. M. P. Boldin, I. L. Mett, E. E. Varfolomeev, I. Chumakov, Y. Shemer-Avni, J. H. Camonis, D. Wallach, Self-association of the "Death Domains" of the p55 tumor necrosis factor (TNF) receptor and Fas/APO1 prompts signaling for TNF and Fas/APO1 effects. *J. Biol. Chem.* **270**, 387–391 (1995).

24. A. Caminero, M. Comabella, X. Montalban, Tumor necrosis factor alpha (TNF-α), anti-TNF-α and demyelination revisited: An ongoing story. *J. Neuroimmunol.* **234**, 1–6 (2011).

25. A. Caminero, M. Comabella, X. Montalban, Role of tumour necrosis factor (TNF)-α and *TNFRSF1A* R92Q mutation in the pathogenesis of TNF receptor-associated periodic syndrome and multiple sclerosis. *Clin. Exp. Immunol.* **166**, 338–345 (2011).

26. F. C. Kimberley, A. A. Lobito, R. M. Siegel, G. R. Screaton, Falling into TRAPS – receptor misfolding in the TNF receptor 1-associated periodic fever syndrome. *Arthritis Res. Ther.* **9**, 217 (2007).

27. G.-M. Deng, L. Zheng, F. K.-M. Chan, M. Lenardo, Amelioration of inflammatory arthritis by targeting the pre-ligand assembly domain of tumor necrosis factor receptors. *Nat. Med.* **11**, 1066–1072 (2005).

28. F. Hadziselimovic, L. R. Emmons, H. Gallati, Soluble tumour necrosis factor receptors p55 and p75 in the urine monitor disease activity and the efficacy of treatment of inflammatory bowel disease. *Gut* **37**, 260–263 (1995).

29. A. A. Lobito, F. C. Kimberley, J. R. Muppidi, H. Komarow, A. J. Jackson, K. M. Hull, D. L. Kastner, G. R. Screaton, R. M. Siegel, Abnormal disulfide-linked oligomerization results in ER retention and altered signaling by TNFR1 mutants in TNFR1-associated periodic fever syndrome (TRAPS). *Blood* **108**, 1320–1327 (2006).

30. Y.-F. Chen, P. Jobanputra, P. Barton, S. Jowett, S. Bryan, W. Clark, A. Fry-Smith, A. Burls, A systematic review of the effectiveness of adalimumab, etanercept and infliximab for the treatment of rheumatoid arthritis in adults and an economic evaluation of their cost-effectiveness. *Health Technol. Assess.* **10**, 10.3310/hta10420 (2006).

31. H. Wajant, Principles of antibody-mediated TNF receptor activation. *Cell Death Differ.* **22**, 1727–1741 (2015).

32. X. Yu, S. James, J. H. Felce, B. Kellermayer, D. A. Johnston, H. T. C. Chan, C. A. Penfold, J. Kim, T. Inzhelevskaya, C. I. Mockridge, Y. Watanabe, M. Crispin, R. R. French, P. J. Duriez, L. R. Douglas, M. J. Glennie, M. S. Cragg, TNF receptor agonists induce distinct receptor clusters to mediate differential agonistic activity. *Commun. Biol.* **4**, 772 (2021).

33. N. Weinelt, C. Karathanasis, S. Smith, J. Medler, S. Malkusch, S. Fulda, H. Wajant, M. Heilemann, S. J. L. van Wijk, Quantitative single-molecule imaging of TNFR1 reveals zafirlukast as antagonist of TNFR1 clustering and TNFα-induced NF-κB signaling. *J. Leukoc. Biol.* **109**, 363–371 (2021).

34. C. H. Lo, N. Vunnam, A. K. Lewis, T.-L. Chiu, B. E. Brummel, T. M. Schaaf, B. D. Grant, P. Bawaskar, D. D. Thomas, J. N. Sachs, An innovative high-throughput screening approach for discovery of small molecules that inhibit TNF receptors. *SLAS Discov.* **22**, 950–961 (2017).

35. S. Ghosh, S. Saha, D. Goswami, S. Bilgrami, S. Mayor, Dynamic imaging of homo-FRET in live cells by fluorescence anisotropy microscopy. *Methods Enzymol.* **505**, 291–327 (2012).

36. J. R. Lakowicz, *Principles of Fluorescence Spectroscopy* (2006), pp. 1–954.

37. S. Ganguly, A. H. A. Clayton, A. Chattopadhyay, Organization of higher-order oligomers of the serotonin$_{1A}$ receptor explored utilizing homo-FRET in live cells. *Biophys. J.* **100**, 361–368 (2011).

38. P. Sharma, R. Varma, R. C. Sarasij, K. Ira, G. Gousset, M. Krishnamoorthy, S. M. Rao, Nanoscale organization of multiple GPI-anchored proteins in living cell membranes. *Cell* **116**, 577–589 (2004).

39. Z. J. Chen, L. J. Sun, Nonproteolytic functions of ubiquitin in cell signaling. *Mol. Cell* **33**, 275–286 (2009).

40. I. E. Wertz, V. M. Dixit, Regulation of death receptor signaling by the ubiquitin system. *Cell Death Differ.* **17**, 14–24 (2010).

41. A. T. Ting, M. J. M. Bertrand, More to life than NF-κB in TNFR1 signaling. *Trends Immunol.* **37**, 535–545 (2016).

42. T. Ha, T. Enderle, D. F. Ogletree, D. S. Chemla, P. R. Selvin, S. Weiss, Probing the interaction between two single molecules: Fluorescence resonance energy transfer between a single donor and a single acceptor. *Proc. Natl. Acad. Sci. U.S.A.* **93**, 6264–6268 (1996).

43. J. Hummert, K. Yserentant, T. Fink, J. Euchner, Y. X. Ho, S. A. Tashev, D.-P. Herten, Photobleaching step analysis for robust determination of protein complex stoichiometries. *Mol. Biol. Cell* **32**, ar35 (2021).

44. M. S. Dietz, D. Haße, D. M. Ferraris, A. Göhler, H. H. Niemann, M. Heilemann, Single-molecule photobleaching reveals increased MET receptor dimerization upon ligand binding in intact cells. *BMC Biophys.* **6**, 6 (2013).

45. E. Macia, M. Ehrlich, R. Massol, E. Boucrot, C. Brunner, T. Kirchhausen, Dynasore, a cell-permeable inhibitor of dynamin. *Dev. Cell* **10**, 839–850 (2006).

46. P. Biswas, P. Roy, S. Jana, D. Ray, J. Das, B. Chaudhuri, R. R. Basunia, B. Sinha, D. K. Sinha, Exploring the role of macromolecular crowding and TNFR1 in cell volume control. *eLife* **13**, e92719 (2024).

47. N. Vunnam, M. Yang, C. H. Lo, C. Paulson, W. D. Fiers, E. Huber, M. Been, D. M. Ferguson, J. N. Sachs, Zafirlukast Is a Promising Scaffold for Selectively Inhibiting TNFR1 Signaling. *ACS Bio Med Chem Au* **3**, 270–282 (2023).

48. H. Wajant, P. Scheurich, TNFR1-induced activation of the classical NF-κB pathway. *FEBS J.* **278**, 862–876 (2011).

49. J. S. Kelloway, Zafirlukast: The First Leukotreene-Receptor Antagonist Approved for the Treatment of Asthma. *Ann. Pharmacother.* **31**, 1012–1021 (1997).

50. E. Sitarska, A. Diz-Muñoz, Pay attention to membrane tension: Mechanobiology of the cell surface. *Curr. Opin. Cell Biol.* **66**, 11–18 (2020).

51. M. Simunovic, E. Evergren, I. Golushko, C. Prévost, H.-F. Renard, L. Johannes, H. T. McMahon, V. Lorman, G. A. Voth, P. Bassereau, How curvature-generating proteins build scaffolds on membrane nanotubes. *Proc. Natl. Acad. Sci. U.S.A.* **113**, 11226–11231 (2016).

52. M. Simunovic, G. A. Voth, Membrane tension controls the assembly of curvature-generating proteins. *Nat. Commun.* **6**, 7219 (2015).

53. A. Colom, E. Derivery, S. Soleimanpour, C. Tomba, M. D. Molin, N. Sakai, M. González-Gaitán, S. Matile, A. Roux, A fluorescent membrane tension probe. *Nat. Chem.* **10**, 1118–1125 (2018).

54. C. Roffay, G. Molinard, K. Kim, M. Urbanska, V. Andrade, V. Barbarasa, P. Nowak, V. Mercier, J. García-Calvo, S. Matile, R. Loewith, A. Echard, J. Guck, M. Lenz, A. Roux, Passive coupling of membrane tension and cell volume during active response of cells to osmosis. *Proc. Natl. Acad. Sci. U.S.A.* **118**, e2103228118 (2021).

55. A. Bhattacharya, M. Agarwal, R. Mukherjee, P. Sen, D. K. Sinha, 3D micro-environment regulates NF-κβ dependent adhesion to induce monocyte differentiation. *Cell Death Dis.* **9**, 914 (2018).

56. G. Kustermans, J. El Benna, J. Piette, S. Legrand-Poels, Perturbation of actin dynamics induces NF-κB activation in myelomonocytic cells through an NADPH oxidase-dependent pathway. *Biochem. J.* **387**, 531–540 (2005).

57. M. Agarwal, P. Biswas, A. Bhattacharya, D. K. Sinha, Reactive oxygen species-mediated cytoplasmic stiffening impairs the phagocytic ability of the macrophage. *J. Cell Sci.* **133**, jcs236471 (2020).

58. V. H. Guaiquil, J. C. Vera, D. W. Golde, Mechanism of Vitamin C Inhibition of Cell Death Induced by Oxidative Stress in Glutathione-depleted HL-60 Cells. *J. Biol. Chem.* **276**, 40955–40961 (2001).

59. D. Ray, D. K. Sinha, Dynamic crosstalk between cytoskeletal filaments regulates dorsoventral cytoplasmic mechanics. *J. Cell Sci.* **138**, JCS263464 (2025).

60. D. F. Legler, O. Micheau, M.-A. Doucey, J. Tschopp, C. Bron, Recruitment of TNF receptor 1 to lipid rafts is essential for TNFα-mediated NF-κB activation. *Immunity* **18**, 655–664 (2003).

61. B. Sinha, D. Köster, R. Ruez, P. Gonnord, M. Bastiani, D. Abankwa, R. V. Stan, G. Butler-Browne, B. Vedie, L. Johannes, N. Morone, R. G. Parton, G. Raposo, P. Sens, C. Lamaze, P. Nassoy, Cells respond to mechanical stress by rapid disassembly of caveolae. *Cell* **144**, 402–413 (2011).

62. S. R. Hubbard, W. T. Miller, Receptor tyrosine kinases: Mechanisms of activation and signaling. *Curr. Opin. Cell Biol.* **19**, 117–123 (2007).

63. X. Su, J. A. Ditlev, E. Hui, W. Xing, S. Banjade, J. Okrut, D. S. King, J. Taunton, M. K. Rosen, R. D. Vale, Phase separation of signaling molecules promotes T cell receptor signal transduction. *Science* **352**, 595–599 (2016).

64. A. Burt, C. K. Cassidy, P. Ames, M. Bacia-Verloop, M. Baulard, K. Huard, Z. Luthey-Schulten, A. Desfosses, P. J. Stansfeld, W. Margolin, J. S. Parkinson, I. Gutsche, Complete structure of the chemosensory array core signalling unit in an *E. coli* minicell strain. *Nat. Commun.* **11**, 743 (2020).

65. A. K. Lewis, C. C. Valley, J. N. Sachs, TNFR1 signaling is associated with backbone conformational changes of receptor dimers consistent with overactivation in the R92Q TRAPS mutant. *Biochemistry* **51**, 6545–6555 (2012).

66. C. C. Valley, A. K. Lewis, D. J. Mudaliar, J. D. Perlmutter, A. R. Braun, C. B. Karim, D. D. Thomas, J. R. Brody, J. N. Sachs, Tumor necrosis factor-related apoptosis-inducing ligand (TRAIL) induces death receptor 5 networks that are highly organized. *J. Biol. Chem.* **287**, 21265–21278 (2012).

67. H. Chakraborty, A. Chattopadhyay, Excitements and challenges in GPCR oligomerization: Molecular insight from FRET. *ACS Chem. Nerosci.* **6**, 199–206 (2015).

68. C. A. Coomer, I. Carlon-Andres, M. Iliopoulou, M. L. Dustin, E. B. Compeer, A. A. Compton, S. Padilla-Parra, Single-cell glycolytic activity regulates membrane tension and HIV-1 fusion. *PLOS Pathog.* **16**, e1008359 (2020).

69. A. Biswas, P. Kashyap, S. Datta, T. Sengupta, B. Sinha, Cholesterol Depletion by MβCD Enhances Cell Membrane Tension and Its Variations-Reducing Integrity. *Biophys. J.* **116**, 1456–1468 (2019).

70. F. Christian, E. Smith, R. Carmody, The regulation of NF-κB subunits by phosphorylation. *Cells* **5**, 12 (2016).

71. Y. Teijeiro-Gonzalez, A. Crnjar, A. J. Beavil, R. L. Beavil, J. Nedbal, A. Le Marois, C. Molteni, K. Suhling, Time-resolved fluorescence anisotropy and molecular dynamics analysis of a novel GFP homo-FRET dimer. *Biophys. J.* **120**, 254–269 (2021).

72. T. Mandal, A. Biswas, T. Ghosh, S. Manikandan, A. Kundu, A. Banerjee, D. Mitra, B. Sinha, Mechano-regulation by clathrin pit-formation and passive cholesterol-dependent tubules during de-adhesion. *Cell. Mol. Life Sci.* **81**, 43 (2024).

73. A. Biswas, A. Alex, B. Sinha, Mapping cell membrane fluctuations reveals their active regulation and transient heterogeneities. *Biophys. J.* **113**, 1768–1781 (2017).

74. A. Mascret, H. Mouhsine, G. Attia, D. Cabrera, M. Benchekroun, P. Gizzi, C. Zerrouki, N. Fourati, J.-F. Zagury, M. S.-I. Veitía, M. Port, New contributions to the drug profile of TNFα inhibitor SPD304: Affinity, selectivity and ADMET considerations. *Eur. J. Pharmacol.* **907**, 174285 (2021).

75. M. M. He, A. S. Smith, J. D. Oslob, W. M. Flanagan, A. C. Braisted, A. Whitty, M. T. Cancilla, J. Wang, A. A. Lugovskoy, J. C. Yoburn, A. D. Fung, G. Farrington, J. K. Eldredge, E. S. Day, L. A. Cruz, T. G. Cachero, S. K. Miller, J. E. Friedman, I. C. Choong, B. C. Cunningham, Small-molecule inhibition of TNF-α. *Science* **310**, 1022–1025 (2005).

76. P. H. Carter, P. A. Scherle, J. A. Muckelbauer, M. E. Voss, R.-Q. Liu, L. A. Thompson, A. J. Tebben, K. A. Solomon, Y. C. Lo, Z. Li, P. Strzemienski, G. Yang, N. Falahatpisheh, M. Xu, Z. Wu, N. A. Farrow, K. Ramnarayan, J. Wang, D. Rideout, V. Yalamoori, P. Domaille, D. J. Underwood, J. M. Trzaskos, S. M. Friedman, R. C. Newton, C. P. Decicco, Photochemically enhanced binding of small molecules to the tumor necrosis factor receptor-1 inhibits the binding of TNF-α. *Proc. Natl. Acad. Sci. U.S.A.* **98**, 11879–11884 (2001).

77. J. O’Connell, J. Porter, B. Kroeplien, T. Norman, S. Rapecki, R. Davis, D. McMillan, T. Arakaki, A. Burgin, D. Fox III, T. Ceska, F. Lecomte, A. Maloney, A. Vugler, B. Carrington, B. P. Cossins, T. Bourne, A. Lawson, Small molecules that inhibit TNF signalling by stabilising an asymmetric form of the trimer. *Nat. Commun.* **10**, 5795 (2019).

78. R. Murali, X. Cheng, A. Berezov, X. Du, A. Schön, E. Freire, X. Xu, Y. H. Chen, M. I. Greene, Disabling TNF receptor signaling by induced conformational perturbation of tryptophan-107. *Proc. Natl. Acad. Sci. U.S.A.* **102**, 10970–10975 (2005).

79. R. K. Rowe, J. L. Harrison, H. Zhang, A. D. Bachstetter, D. P. Hesson, B. F. O'Hara, M. I. Greene, J. Lifshitz, Novel TNF receptor-1 inhibitors identified as potential therapeutic candidates for traumatic brain injury. *J. Neuroinflammation* **15**, 154 (2018).

80. F. Mancini, C. M. Toro, M. Mabilia, M. Giannangeli, M. Pinza, C. Milanese, Inhibition of tumor necrosis factor-α (TNF-α)/ TNF-α receptor binding by structural analogues of suramin. *Biochem. Pharmacol.* **58**, 851–859 (1999).

81. R. Alzani, A. Corti, L. Grazioli, E. Cozzi, P. Ghezzi, F. Marcucci, Suramin induces deoligomerization of human tumor necrosis factor alpha. *J. Biol. Chem.* **268**, 12526–12529 (1993).

82. Q.-L. Hu, S. H. Liang, Novel cyclic peptides inhibiting tumor necrosis factor receptor 1 activity. *ACS Med. Chem. Lett.* **16**, 363–365 (2025).
